# Anti‐Inflammatory Effects of *Hermetia illucens* Fermented With Riboflavin‐Producing *Leuconostoc mesenteroides* KCCM13067P in RAW 264.7 Cells and Colitis Mouse Model

**DOI:** 10.1002/fsn3.71763

**Published:** 2026-04-09

**Authors:** Seok Jun Son, Ah‐Ram Han, Mi Jeong Sung, Hyun‐jin Na, Sun Mee Hong, Sang‐Hee Lee

**Affiliations:** ^1^ Korea Food Research Institute Iseo‐myeon Jeollabuk‐do Republic of Korea; ^2^ Division of Food Biotechnology University of Science and Technology Daejeon Republic of Korea; ^3^ Institute of Natural BIO Industry for Namwon Namwon‐si Jeollabuk‐do Republic of Korea

**Keywords:** anti‐inflammatory activity, gut microbiota, *Hermetia illucens*, inflammatory bowel disease, *Leuconostoc mesenteroides*, riboflavin

## Abstract

Fermentation is widely used to enhance the nutritional and functional properties of food substrates; however, the biological effects of insect‐derived materials fermented with riboflavin‐producing lactic acid bacteria remain largely unexplored. This study investigated the anti‐inflammatory potential of *Hermetia illucens* hot‐water extract fermented with *Leuconostoc mesenteroides* KCCM13067P (HeHi_Lm) using LPS‐stimulated RAW 264.7 macrophages and a dextran sulfate sodium (DSS)‐induced chronic colitis mouse model. Fermentation significantly increased vitamin B_2_ derivatives, with riboflavin, flavin adenine dinucleotide (FAD), and flavin mononucleotide (FMN) increasing from 7.82, 10.58, and 2.60 μg/g to 18.53, 29.84, and 9.29 μg/g, respectively, representing an approximately 2.7‐fold increase in total vitamin B_2_ content. Fermentation also enriched free amino acids and bioactive amines, including glycine, alanine, cystathionine, γ‐aminobutyric acid, and ethanolamine. In vitro, HeHi_Lm significantly suppressed LPS‐induced nitric oxide production and reduced IL‐6 secretion, whereas TNF‐α reduction was not statistically significant. In vivo, HeHi_Lm administration alleviated DSS‐induced colitis symptoms, as demonstrated by improved body weight recovery, reduced disease activity index scores, and attenuation of colon shortening. Histopathological damage and spleen and liver weight/body weight ratios were also improved. Furthermore, HeHi_Lm significantly reduced colonic expression of inflammatory mediators, including iNOS, COX‐2, IL‐6, and IL‐1β. Gut microbiota analysis revealed compositional shifts characterized by increased Firmicutes and Bacteroidetes and reduced *Verrucomicrobia*, with enrichment of Lactobacillaceae and Oscillospiraceae. These findings indicate that fermentation of *H. illucens* with *L. mesenteroides* enhances functional metabolite profiles and is associated with reduced inflammatory responses and microbiota restructuring in experimental colitis, supporting its potential as a sustainable functional food ingredient.

## Graphical abstract

Fermentation of *Hermetia illucens* with riboflavin‐producing *Leuconostoc mesenteroides* KCCM13067P enhanced vitamin B_2_ derivatives and bioactive metabolites, including amino acids and amines. The fermented product (HeHi_Lm) suppressed inflammatory responses in LPS‐stimulated RAW 264.7 macrophages and alleviated DSS‐induced colitis in mice. These effects were associated with reduced inflammatory mediators and cytokines, as well as beneficial modulation of gut microbiota composition. These findings support the potential of fermented insect‐derived substrates as sustainable functional food ingredients.

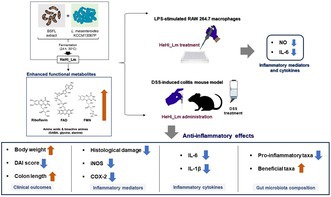

## 1 Introduction

Inflammatory bowel diseases (IBDs), primarily Crohn's disease and ulcerative colitis (UC), are characterized by acute and chronic intestinal inflammation with a multifactorial etiology (Chassaing et al. 2014). Patients experience severe gastrointestinal symptoms, including diarrhea, abdominal pain, bleeding, anemia, and weight loss. IBD pathogenesis involves complex interactions among environmental and genetic factors, immune dysregulation, gut barrier dysfunction, and alterations in the gut microbiome, although the precise mechanisms remain incompletely understood (Cuzzocrea et al. 2001; Fiocchi 1998; Macfarlane et al. 2005). To investigate disease pathogenesis and therapeutic strategies, various animal models have been developed, among which the dextran sulfate sodium (DSS)–induced colitis model is widely used because of its simplicity, reproducibility, and experimental controllability (Chassaing and Darfeuille–Michaud 2011).

Black soldier fly ( *Hermetia illucens* ; Hi) larvae (BSFL) are saprophytic insects that utilize organic wastes such as food waste, animal manure, and agricultural by‐products (Tomberlin and Van Huis 2020; Zheng et al. 2012). Due to their high protein and lipid contents, BSFL have attracted interest as alternative protein sources for food and feed applications (Erickson et al. 2004; Purba et al. 2020). *H. illucens* is increasingly recognized as a sustainable edible insect with potential for large‐scale and hygienic production. In the European Union, edible insects are regulated as novel foods under Regulation (EU) 2015/2283, administered by the European Commission with scientific risk assessment conducted by the European Food Safety Authority (EFSA). Although *H. illucens* has not yet been formally authorized for human consumption in most regulatory jurisdictions, including the EU, this reflects the ongoing regulatory evaluation process rather than safety concerns (Liguori et al. 2022; Wildbacher et al. 2025). As part of the novel food approval process, comprehensive compositional, microbiological, toxicological, and allergenicity data have been submitted for dried whole‐larvae products (“Hermetia meal”) (Meijer et al. 2025). In addition, BSFL‐based high‐protein foods have already been developed and evaluated in several regions, including Southeast Asia (Wrasiati et al. 2025). Together with their high nutritional value and feasibility for hygienic, large‐scale production, these findings support *H. illucens* as a promising edible insect under regulatory evaluation (da‐Silva et al. 2024).

*L. mesenteroides* is a heterolactic fermentative lactic acid bacterium widely used in food fermentation, particularly in dairy and vegetable products (Özcan et al. 2019; Dols et al. 1997; Jung et al. 2012). Probiotic LAB, including *Lactobacillus* and *Bifidobacterium* species as well as *L. mesenteroides* , have been reported to ameliorate IBD by modulating cytokine production, immune responses, epithelial integrity, and gut microbiota composition (Gibson et al. 2017; Hudson et al. 2017; Sanders et al. 2019). Several *L. mesenteroides* strains have shown therapeutic potential in IBD models by reducing disease activity and suppressing pro‐inflammatory mediators (Moon et al. 2023; Kuda et al. 2014). In the present study, a riboflavin‐producing strain, *L. mesenteroides* KCCM13067P, was selected based on its functional relevance and metabolic capability for fermentation. Lactic acid bacteria are known to efficiently ferment diverse organic substrates and generate functional metabolites that can enhance the nutritional and bioactive properties of fermented products (Zarour et al. 2024). Accordingly, *L. mesenteroides* KCCM13067P was selected as a suitable for fermenting *H. illucens* –derived substrates in this study, as previous studies have demonstrated that insect biomass, including *H. illucens* , can serve as an effective substrate for lactic acid bacterial fermentation (Hadj Saadoun et al. 2020). Fermentation with riboflavin‐producing lactic acid bacteria may further enhance the functional value of insect‐derived substrates through the combined effects of microbial metabolites and nutrient biotransformation, providing a potential synergistic strategy for developing functional food ingredients. Notably, previous studies have investigated either insect‐derived materials or *L. mesenteroides* fermentation independently; however, insect‐derived substrates fermented with a riboflavin‐producing *L. mesenteroides* strain have not previously been evaluated in inflammatory cell or animal models. Therefore, this study aimed to investigate the anti‐inflammatory effects of *H. illucens* fermented with the riboflavin‐producing strain *L. mesenteroides* KCCM13067P using RAW 264.7 macrophages and a DSS‐induced colitis mouse model, with particular emphasis on the combined functional contributions of insect‐derived substrates and fermentation‐derived metabolites.

Macrophages are key regulators of intestinal inflammation, and their excessive activation in colitis is associated with increased production of pro‐inflammatory cytokines, including TNF‐α and IL‐6, as well as nitric oxide (NO), leading to tissue damage (Huang 2021; Navegantes et al. 2017). Riboflavin, a precursor of flavin adenine dinucleotide (FAD) and flavin mononucleotide (FMN), modulates oxidative and inflammatory responses by regulating NADPH oxidase activity, reactive oxygen species (ROS) production, and NF‐κB signaling (Hashida et al. 2004; Pisoschi et al. 2022). In addition, riboflavin has been reported to limit excessive granulocyte infiltration and inflammatory activation (Verdrengh and Tarkowski 2005; Hashida et al. 2004; Suzuki et al. 1995; Qureshi et al. 2011). Importantly, *L. mesenteroides* is capable of synthesizing riboflavin with reported anti‐inflammatory potential (Levit et al. 2017; Thakur et al. 2016), yet its application in IBD has not been explored.

Gut microbial composition is a central determinant of intestinal inflammation, as dysbiosis disrupts epithelial barrier function and skews mucosal immune responses toward pro‐inflammatory states (Kennedy and Chang 2025). Microbiota‐driven alterations in intestinal metabolites regulate epithelial integrity, oxidative stress, and immune cell polarization, while diet–microbiome–metabolite interactions can amplify inflammatory signaling in susceptible hosts (Ning et al. 2023; Pereira et al. 2024). Accordingly, favorable shifts in gut microbiota are understood as compositional and functional changes that support barrier integrity, promote immunoregulation, and normalize inflammation‐associated microbial metabolites.

Collectively, these findings suggest that fermentation of *H. illucens* with riboflavin‐producing lactic acid bacteria may represent a promising approach for generating functionally enhanced insect‐derived materials, potentially involving changes in fermentation‐derived metabolites and gut microbiota‐associated responses. Building on this background, the present study hypothesized that fermentation of *H. illucens* with *L. mesenteroides* enhances its immunomodulatory potential via combined nutritional and microbial interactions rather than a single defined molecular mechanism. To test this hypothesis, we investigated the anti‐inflammatory effects of *H. illucens* fermented with *L. mesenteroides* using LPS‐stimulated RAW 264.7 macrophages in vitro and a DSS‐induced colitis mouse model in vivo.

## 2 Materials and Methods

### 2.1 Materials

Black soldier fly larvae ( *H. illucens* ) powder was obtained from a commercial supplier (SignalCare, Cheongdo, Republic of Korea). Dextran sulfate sodium (DSS; molecular weight 36,000–50,000 Da) was purchased from MP Biomedicals (Santa Ana, CA, USA). Lipopolysaccharide (LPS), riboflavin, flavin mononucleotide (FMN), and flavin adenine dinucleotide (FAD) analytical standards were obtained from Sigma‐Aldrich (St. Louis, MO, USA). RAW 264.7 murine macrophages were obtained from the Korea Cell Line Bank (Seoul, Republic of Korea). ELISA kits for TNF‐α and IL‐6 were purchased from R&D Systems (Minneapolis, MN, USA). All other reagents were of analytical grade unless otherwise specified.

### 2.2 Preparation of HeHi and Fermented HeHi_Lm

A riboflavin‐producing *L. mesenteroides* strain (KCCM13067P) was previously isolated from a fish farm by the National Institute of Fisheries Science (Pohang, Republic of Korea) (Son et al. 2023). The strain used in this study, *L. mesenteroides* KCCM13067P, was deposited at the Korean Culture Center of Microorganisms (KCCM), an internationally recognized public culture collection, ensuring strain traceability and accessibility. Black soldier fly larvae (BSFL) powder was prepared by drying, milling, and defatting processes. The powdered *H. illucens* was suspended in distilled water at 5% (w/v) and sterilized at 121°C for 15 min using an autoclave (PAC‐100; U1TECH, Suwon, Republic of Korea) to obtain the hot‐water extract (HeHi). Unfermented HeHi was used as the control. Fermentation was conducted by inoculating *L. mesenteroides* (1 × 10^6^ CFU/mL) into the prepared HeHi substrate (1%, w/w or w/v), followed by incubation under oxygen‐limited conditions at 30°C for 24 h. The fermentation duration (24 h) was selected based on preliminary experiments showing that viable cell counts reached a maximum at approximately 24 h and remained stable thereafter, indicating no further substantial increase in microbial growth with extended incubation, which is consistent with previous reports that lactic acid bacteria including *L. mesenteroides* typically reach peak cell density around 24 h of fermentation (Kim and Han 2019). After fermentation, fermented HeHi (HeHi_Lm) and unfermented HeHi were centrifuged at 3000 × g for 10 min, and the collected supernatants were subsequently freeze‐dried to obtain lyophilized powders. The resulting lyophilized powders were stored at −20°C and reconstituted in sterile distilled water at the required concentrations prior to further in vitro and in vivo analyses.

### 2.3 Determination of Vitamin B_2_ Derivatives by HPLC

Quantification of riboflavin, FMN, and FAD was performed using high performance liquid chromatography (HPLC) according to a previously described method (Son et al. 2023). Supernatants were filtered prior to analysis. Analytical standards were prepared in 0.5% (w/v) methanol. Chromatographic separation was achieved using a Capcell Pak UG120 C18 column (4.6 × 250 mm, 5 μm; Osaka Soda, Osaka, Japan) maintained at 40°C. The mobile phase consisted of methanol and 10 mM NaH_2_PO₄ buffer (pH 5.5) at a ratio of 35:65 (v/v). The injection volume was 10 μL, the flow rate was 0.8 mL/min, and detection wavelengths were set at 445 and 530 nm. Samples were stored at −70°C until analysis.

### 2.4 Cell Culture

RAW 264.7 macrophages were cultured in Dulbecco's modified Eagle's medium (DMEM; Thermo Fisher Scientific, Waltham, MA, USA) supplemented with 10% fetal bovine serum and 1% penicillin–streptomycin. Cells were maintained at 37°C in a humidified incubator containing 5% CO_2_.

### 2.5 Nitric Oxide (NO) Quantification

RAW 264.7 cells were seeded into 96‐well plates at a density of 2 × 10^4^ cells/well and incubated for 24 h. Cells were pretreated with HeHi_Lm (100, 400, or 800 μg/mL) for 2 h and subsequently stimulated with LPS (1 μg/mL) for 24 h. Cell culture supernatants were mixed (1:1) with Griess reagent containing 1% sulfanilamide, 0.1% N‐(1‐naphthyl)ethylenediamine dihydrochloride, and 2.5% phosphoric acid, followed by incubation for 20 min in the dark. Absorbance was measured at 540 nm using a microplate reader (Tecan, Männedorf, Switzerland), and nitrite levels were expressed relative to untreated control cells.

### 2.6 Cytokine Quantification

RAW 264.7 cells were seeded at 2 × 10^4^ cells/well and incubated for 24 h. Cells were pretreated with HeHi_Lm for 2 h followed by LPS stimulation (1 μg/mL) for 24 h. Supernatants were collected and analyzed for TNF‐α and IL‐6 concentrations using ELISA kits according to the manufacturer's instructions.

### 2.7 Animals and Experimental Design

Male C57BL/6 mice (7 weeks old) were obtained from Orient Bio (Seongnam, Republic of Korea). Animals were maintained under controlled conditions (20°C ± 3°C, 50% ± 20% humidity, 12 h light/dark cycle) with free access to food and reverse‐osmosis water. After 1 week of acclimatization, mice were randomly assigned using a random allocation procedure to four groups (*n* = 4–5 per group): normal control (NC), DSS control, low‐dose HeHi_Lm (1000 mg/kg), and high‐dose HeHi_Lm (2000 mg/kg). HeHi_Lm was administered orally once daily. The initial sample size was *n* = 6 per group; however, the final number of animals included in the analysis was *n* = 4–5 per group due to the application of humane endpoint criteria in DSS‐treated animals. Primary outcome measures included body weight change, disease activity index (DAI), colon length, histopathological evaluation, and inflammatory marker expression. All experimental procedures were approved by the Institutional Animal Care and Use Committee of the Korea Food Research Institute (KFRI‐M‐22032). This study was conducted and reported in accordance with the ARRIVE 2.0 guidelines.

### 2.8 Induction of DSS Colitis and Disease Activity Index (DAI)

Chronic colitis was induced by administering 2% (w/v) DSS in drinking water in three cycles (days 1–5, 11–13, and 21–23), with reverse‐osmosis water provided during recovery periods (Lee et al. 2016) (Figure 4A). Disease severity was evaluated daily using the disease activity index (DAI) based on body weight loss, stool consistency, and fecal blood, according to Friedman et al. (2009) (Table 1). Individual scores were summed to obtain a composite DAI value.

**TABLE 1:** Scoring system for the disease activity index (DAI).^a^

Score	Weight loss (%)	Stool consistency	Hematochezia^b^
0	None	Normal	Absence
1	0–10	—	—
2	11–15	Loose feces^c^	
3	16–20	—	
4	> 20	Diarrhea	Presence

^a^ DAI = (weight loss score) + (fecal consistency score) + (hematochezia score).

^b^ Presence of gross blood in the feces or anus.

^c^ Formation of feces that readily become paste on the anus of mice.

### 2.9 Histopathological and Molecular Analyses

Colon tissues were fixed in 10% neutral‐buffered formalin, embedded in paraffin, sectioned, and stained with hematoxylin and eosin (H&E). Histological changes were evaluated under a light microscope (Eclipse 80i; Nikon, Melville, NY, USA). For Western blotting, colon tissues were homogenized in RIPA buffer containing phenylmethanesulfonyl fluoride. Equal amounts of protein (30 μg) were separated by SDS–PAGE and transferred onto PVDF membranes. Membranes were incubated with primary antibodies against iNOS, COX‐2, and β‐actin (Cell Signaling Technology, Danvers, MA, USA), followed by HRP‐conjugated secondary antibodies. Signals were detected using enhanced chemiluminescence and quantified using ImageJ software. Western blot experiments were performed twice independently under identical conditions, and densitometric values represent averages of technical replicates. Total RNA was extracted using the PureLink RNA Mini Kit (Invitrogen, Carlsbad, CA, USA). cDNA synthesis was performed using a commercial reverse transcription premix (iNtRON Biotechnology, Seongnam, Republic of Korea). Quantitative PCR was conducted using Power SYBR Green Master Mix (Applied Biosystems) on a QuantStudio 3 system. Gene expression levels were normalized to β‐actin and GAPDH. Primer sequences are listed in Table 2.

**TABLE 2:** Primer sequences used to detect mRNA specific to target genes.

Gene	Sequence (5′ → 3′)	Accession number
β‐Actin	F: 5′‐CAGCTGAGAGGGAAATCGTG‐3′ R: 5′‐CGTTGCCAATAGTGATGACC‐3′	NM_031144.3
TNF‐α	F: 5′‐ACCCTCACACTCAGATCATC‐3′ R: 5′‐GAGTAGACAAGGTACAACCC‐3′	NM_012675.3
IL 6	F: 5′‐TGGAGTACCATAGCTACCTG‐3′ R: 5′‐TGACTCCAGCTTATCTGTTA‐3′	NM_012589.2
IL‐1β	F: 5′‐TGTAATGAAAGACGGCACAC‐3′ R: 5′‐TCTTCTTTGGGTATTGCTTG‐3′	NM_031512.2

Abbreviations: F, forward primer; IL‐6, interleukin‐6; R, reverse primer; TNF‐α, tumor necrosis factor‐α.

### 2.10 Gut Microbiota Analysis

Fecal samples were collected prior to euthanasia and stored at −80°C. DNA was extracted using the DNeasy PowerSoil Kit (Qiagen). Library preparation targeted the V3–V4 region of the 16S rRNA gene and sequencing was performed on an Illumina MiSeq platform (2 × 300 bp) at Macrogen (Seoul, Republic of Korea). Sequence data were processed using QIIME (v1.9) for taxonomic assignment and relative abundance analysis.

### 2.11 Statistical Analysis

All quantitative data are presented as mean ± standard deviation (SD) based on at least three independent biological replicates unless otherwise stated. Technical replicates, when applicable, were averaged and were not treated as independent samples for statistical analysis. Statistical analyses were performed using one‐way analysis of variance (ANOVA) with GraphPad Prism software (version 8.0; GraphPad Software, La Jolla, CA, USA). Differences were considered statistically significant at *p* < 0.05.

## 3 Results

### 3.1 Vitamin B_2_ Production by *L. mesenteroides*

**FIGURE 1:**
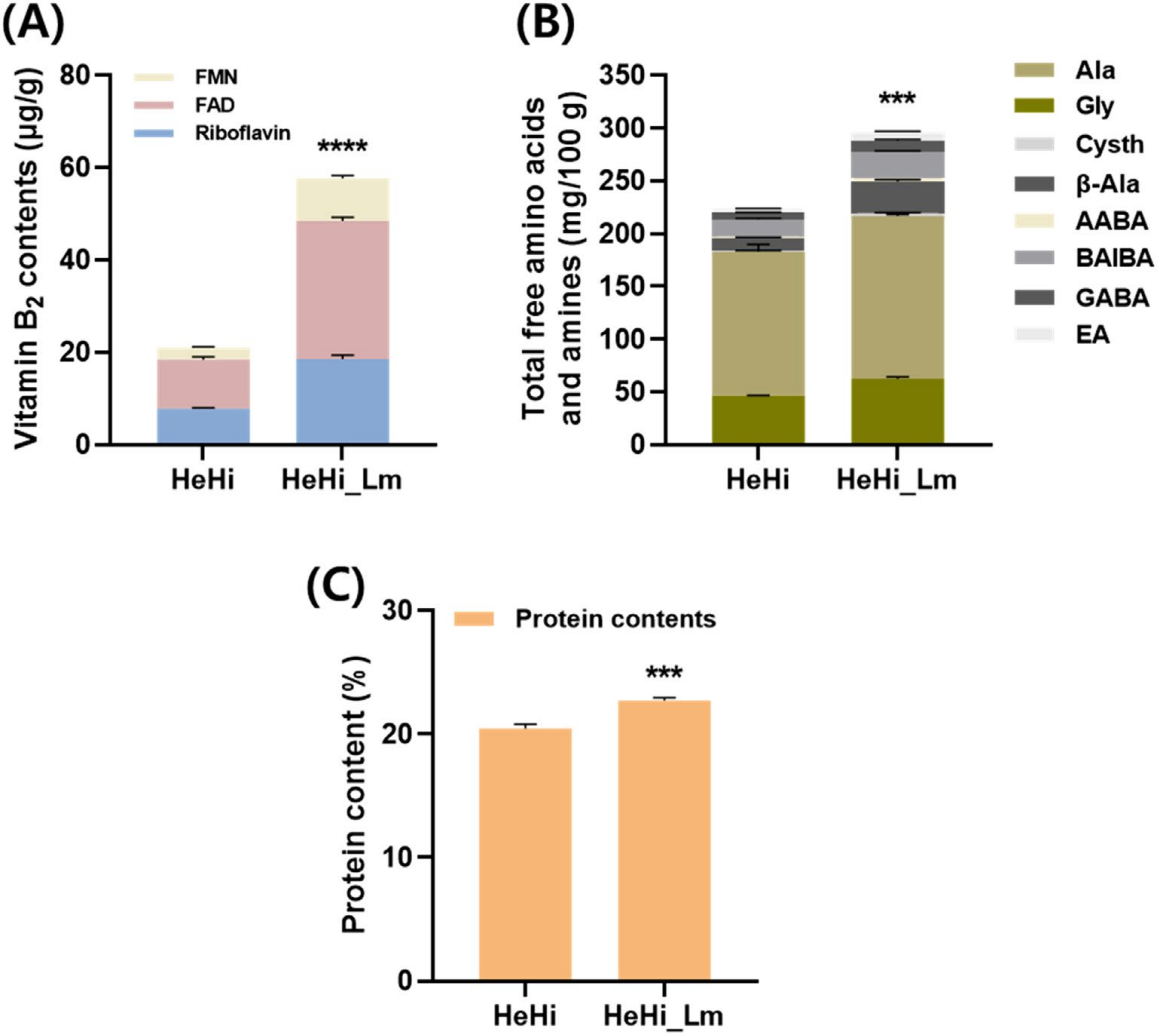
Concentration of riboflavin and fermentation‐derived metabolites produced by *L. mesenteroides* in *H. illucens lar*vae powder. (A) Vitamin B_2_ contents, including riboflavin, flavin adenine dinucleotide (FAD), and flavin mononucleotide (FMN), in HeHi and HeHi_Lm. (B) Total free amino acids and amines in HeHi and HeHi_Lm, expressed as the summed contents of individual components. (C) Protein content (%) of HeHi and HeHi_Lm. Data are presented as mean ± SD (*n* = 3 independent biological replicates per group). ***p < 0.001 and ****p < 0.0001 compared with the HeHi control group. HeHi, *H. illucens ext*racted with hot water (5% v/v); HeHi_Lm, HeHi fermented with *L. mesenteroides. Ab*breviations: FAD, flavin adenine dinucleotide; FMN, flavin mononucleotide; Ala, L‐alanine; Gly, glycine; Cysth, L‐cystathionine; AABA, DL‐2‐aminobutyric acid; β‐Ala, β‐alanine; BAIBA, DL‐3‐aminoisobutyric acid; GABA, 4‐aminobutyric acid; EA, 2‐aminoethanol.

Vitamin B_2_, including riboflavin, FAD, and FMN, produced by *L. mesenteroides* after fermentation for 24 h, was analyzed in HeHi_Lm and compared with that in HeHi. The riboflavin, FAD, and FMN levels in HeHi were 7.82 ± 0.22, 10.58 ± 0.62, and 2.60 ± 0.21 μg/g (total 20.99 ± 0.74 μg/g), respectively, while those in HeHi_Lm were 18.53 ± 1.01, 29.84 ± 1.31, and 9.29 ± 0.61 μg/g (total 57.69 ± 2.93 μg/g), respectively (Figure 1A). The riboflavin, FAD, and FMN contents of HeHi_Lm fermented by *L. mesenteroides* were approximately 2.3‐, 2.8‐, and 3.5‐fold higher, respectively, than those of HeHi. Fermented HeHi_Lm showed a 2.7‐fold increase in vitamin B_2_ content.

### 3.2 Enhanced Production of Free Amino Acids and Amines by *L. mesenteroides* Fermentation

As shown in Figure 1B, fermentation of HeHi with *L. mesenteroides* (HeHi_Lm) resulted in a marked increase in total free amino acids and amines compared with unfermented HeHi. Glycine and alanine levels increased from 46.63 to 61.45 and from 137.9 to 153.53, respectively, and β‐alanine was also elevated following fermentation (1.64 vs. 2.62). A pronounced increase was observed for sulfur‐containing cysthionine, which increased from 12.22 in HeHi to 29.28 in HeHi_Lm. In addition, fermentation enhanced several bioactive amines, including α‐aminobutyric acid, β‐aminoisobutyric acid, γ‐aminobutyric acid, and ethanolamine. Consistent with these compositional changes, total protein content was slightly increased following fermentation, rising from 20.47 ± 0.35 in HeHi to 22.74 ± 0.22 in HeHi_Lm (Figure 1C). Collectively, these results indicate that fermentation with *L. mesenteroides* KCCM13067P enriches free amino acids and related metabolites in HeHi, reflecting enhanced production of low‐molecular‐weight bioactive compounds.

### 3.3 Effects of HeHi_Lm on NO Production in LPS‐Induced RAW 264.7 Cells

**FIGURE 2:**
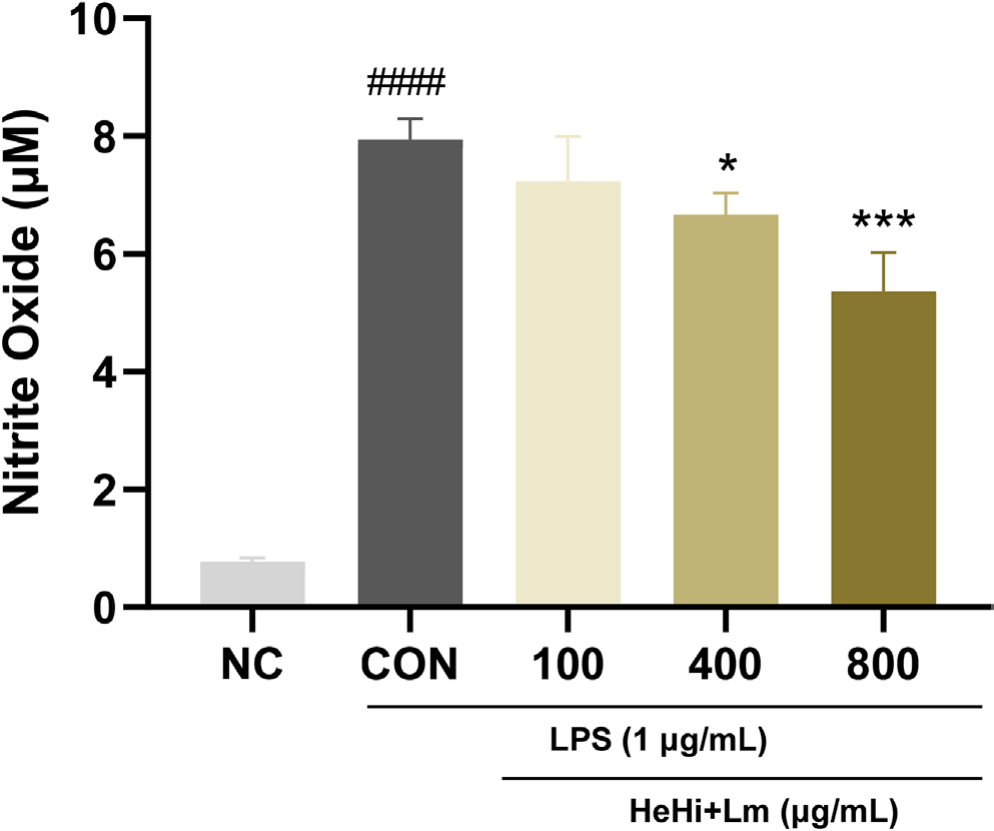
Effects of HeHi_Lm on NO production in LPS‐induced RAW 264.7 cells. Data are presented as mean ± SD (*n* = 3 independent biological experiments). ^####^ *p* < 0.0001 compared with the normal control group (NC). *p < 0.05 and ***p < 0.001 compared with the LPS control group. The “Normal” group represents non‐treated cells, and the “Control” group represents LPS‐treated cells. “100,” “400,” and “800” indicate HeHi_Lm treatment concentrations (100, 400, and 800 μg/mL). LPS, lipopolysaccharide.

RAW 264.7 macrophages were stimulated with LPS (1 μg/mL) to induce an inflammatory response, which significantly increased NO production compared with the normal control group (7.94 μM) (Figure 2). Pretreatment with HeHi_Lm across a concentration range (100–800 μg/mL) reduced LPS‐induced NO production in a concentration‐dependent manner, with significant inhibition observed at 400 and 800 μg/mL. These results indicate that HeHi_Lm suppresses inflammatory NO production in a dose‐dependent fashion.

### 3.4 Effects of HeHi_Lm on Cytokine Production in LPS‐Induced RAW 264.7 Cells

**FIGURE 3:**
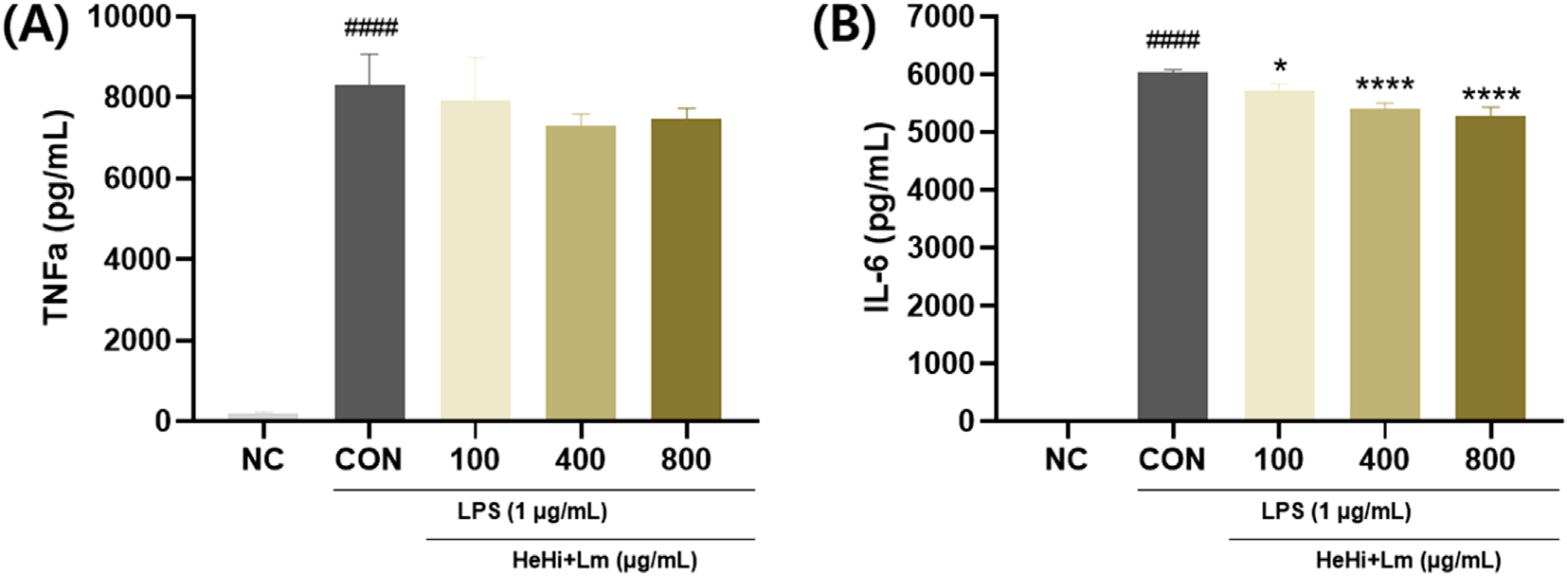
Effects of fermented HeHi_Lm on cytokine production in LPS‐induced RAW 264.7 cells. (A) TNF‐α and (B) IL‐6 levels. Data are presented as mean ± SD (*n* = 3 independent biological experiments). ^####^ *p* < 0.0001 compared with the normal control group (NC). *p < 0.05 and ****p < 0.0001 compared with the LPS control group. The “Normal” group represents non‐treated cells, the “Control” group represents LPS‐treated cells, and “100,” “400,” and “800” indicate HeHi_Lm treatment concentrations (100, 400, and 800 μg/mL). LPS, Lipopolysaccharide.

Cell culture supernatants were analyzed to evaluate the secretion of pro‐inflammatory cytokines. LPS stimulation markedly increased TNF‐α and IL‐6 production compared with the normal control group (8311.33 and 6027.44 pg/mL, respectively) (Figure 3). HeHi_Lm pretreatment reduced TNF‐α levels at 100, 400, and 800 μg/mL (7943.00, 7287.67, and 7465.00 pg/mL, respectively); however, these reductions were not statistically significant (*p* = 0.999, 0.267, and 0.479, respectively). In contrast, IL‐6 secretion was significantly decreased in a concentration‐dependent manner following HeHi_Lm treatment, with levels reduced to 5712.00, 5404.22, and 5291.56 pg/mL at 100, 400, and 800 μg/mL, respectively (Figure 3B). Together, these findings suggest that HeHi_Lm attenuates LPS‐induced cytokine production, particularly IL‐6.

### 3.5 Effect of HeHi_Lm on DSS‐Induced Colitis Symptoms

**FIGURE 4:**
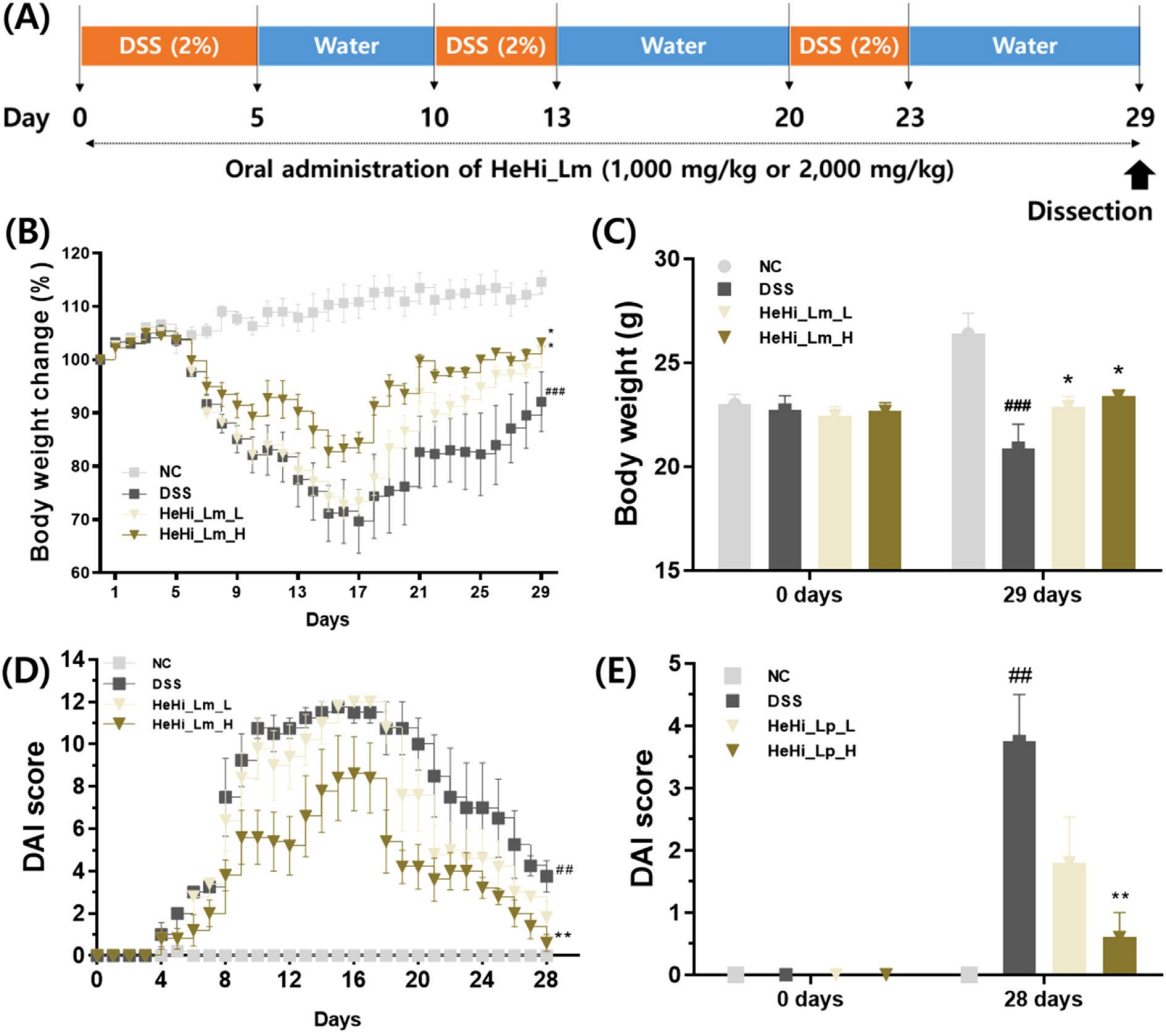
Effects of HeHi_Lm treatment on clinical symptoms in mice with dextran sulfate sodium (DSS)‐induced chronic colitis. (A) Schematic representation of the experimental design and treatment schedule. (B, C) Body weight changes. (D, E) Disease activity index (DAI) scores. Data are presented as mean ± SD (*n* = 4–5 mice per group; biological replicates). ^##^ *p* < 0.01 and ^###^ *p* < 0.001 compared with the normal control group (NC); *p < 0.05 and **p < 0.01 compared with the DSS control group.

To evaluate the protective effects of HeHi_Lm in DSS‐induced mice, we assessed colitis symptoms by monitoring body weight changes and DAI scores in the DSS‐induced mouse colitis model. Three cycles of 2% DSS treatment resulted in three major colitis symptoms: noticeable weight loss, diarrhea, and hematochezia. However, normal mice did not show these symptoms (Figure 4B–E). The DSS‐induced mice treated with HeHi_Lm (low [L] and high [H]) showed lower body weight loss than did the DSS control mice. Additionally, body weight restoration was significantly increased in HeHi_Lm_H‐treated mice compared with that in DSS‐treated mice. On day 29, the weight change was highest at 114.59 ± 4.0% in NC, and in the DSS control group, it was significantly decreased to 92.14 ± 10.7% (*p* < 0.001, Figure 4B,C). In the HeHi_Lm_L group, body weight increased to 101.88 ± 2.2% (*p* < 0.01), and in the HeHi_Lm_H group, body weight significantly increased to 103.25 ± 4.8% (*p* < 0.01).

The DAI score calculated in this study showed a trend conforming to the weight change results, that is, DAI increased with weight loss and decreased with weight gain (Figure 4D,E). In all treated groups, DAI increased and then decreased every three cycles. DAI scoring on day 28, the day before euthanasia, confirmed a significant difference: the score was 0 points for the NC group and 3.75 ± 1.5 points for the DSS control group (*p* < 0.01), 1.8 ± 1.3 points for the HeHi_Lm_L group, and 0.6 ± 0.2 points (*p* < 0.01) for the HeHi_Lm_H group.

### 3.6 Effect of HeHi_Lm on Colon Histopathological Alterations and Spleen and Liver Weight

**FIGURE 5:**
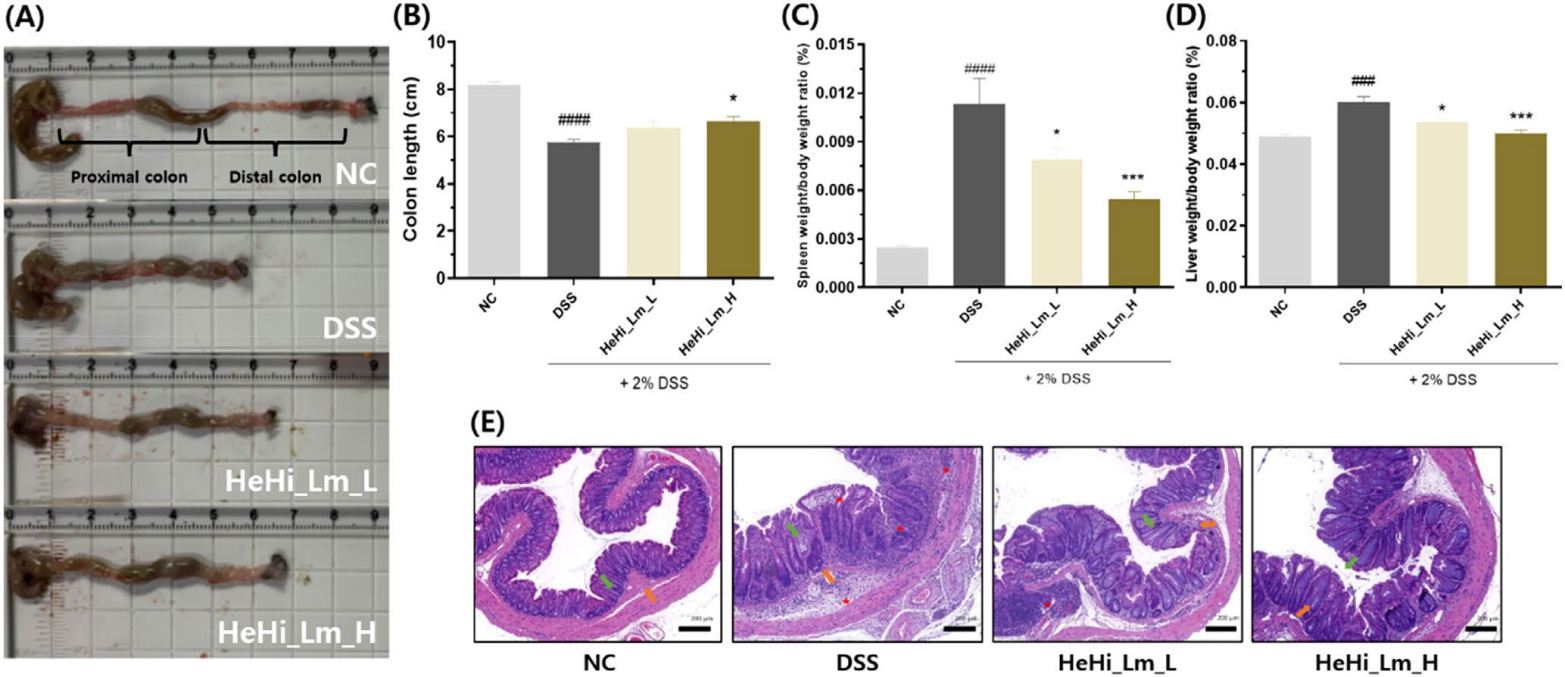
Protective effects of HeHi_Lm against DSS‐induced chronic colitis in mice. (A) Representative colon images from each group after sacrifice. (B) Colon length. (C) Spleen weight/body weight ratio. (D) Liver weight/body weight ratio. (E) Representative hematoxylin and eosin (H&E)‐stained colon sections (scale bar = 200 μm). Red asterisks indicate inflammatory cell infiltration; green arrows indicate columnar epithelial cells; orange arrows indicate crypts. Data are presented as mean ± SD (*n* = 4–5 mice per group; biological replicates). ^###^ *p* < 0.001 and ^####^ *p* < 0.0001 compared with the normal control group (NC); *p < 0.05 and ***p < 0.001 compared with the DSS control group.

We verified the anti‐inflammatory effect of the treatments on colon length, spleen and liver weight, and mucosal integrity in the DSS‐induced murine model because the change in colon length in colitis animal models is associated with the progression of inflammation. Large intestine length in the DSS group (5.75 ± 0.26 cm) was significantly shorter than in the NC group (8.18 ± 0.29 cm, *p* < 0.001, Figure 5A,B); the lengths were 6.38 ± 0.64 and 6.66 ± 0.42 cm (*p* < 0.05) in the HeHi_Lm_L and HeHi_Lm_H groups, respectively.

The spleen weight/body weight ratio of mice with DSS‐induced colitis was significantly higher (*p* < 0.0001) than that of normal mice and significantly lower than in the HeHi_Lm_L (*p* < 0.05) and HeHi_Lm_H groups (*p* < 0.001) (Figure 5C). The liver weight/body weight ratio of mice with DSS‐induced colitis was significantly higher (*p* < 0.001) than that of normal control mice and significantly lower than in the HeHi_Lm_L (*p* < 0.05) and HeHi_Lm_H groups (*p* < 0.001) (Figure 5D).

Histopathological examination of colonic tissue samples revealed no abnormalities in the colonic tissues of the NC group. However, inflammatory cell infiltration, destruction of the nucleus in mucous membranes, and epithelial tissue loss were observed in the DSS control group. The tissues in the HeHi_Lm_L and HeHi_Lm_H groups were partially damaged compared with those in the NC group; however, DSS‐induced damage was ameliorated (Figure 5E).

### 3.7 Effects of HeHi_Lm Treatment on Inflammatory Mediators in Colon Tissue

**FIGURE 6:**
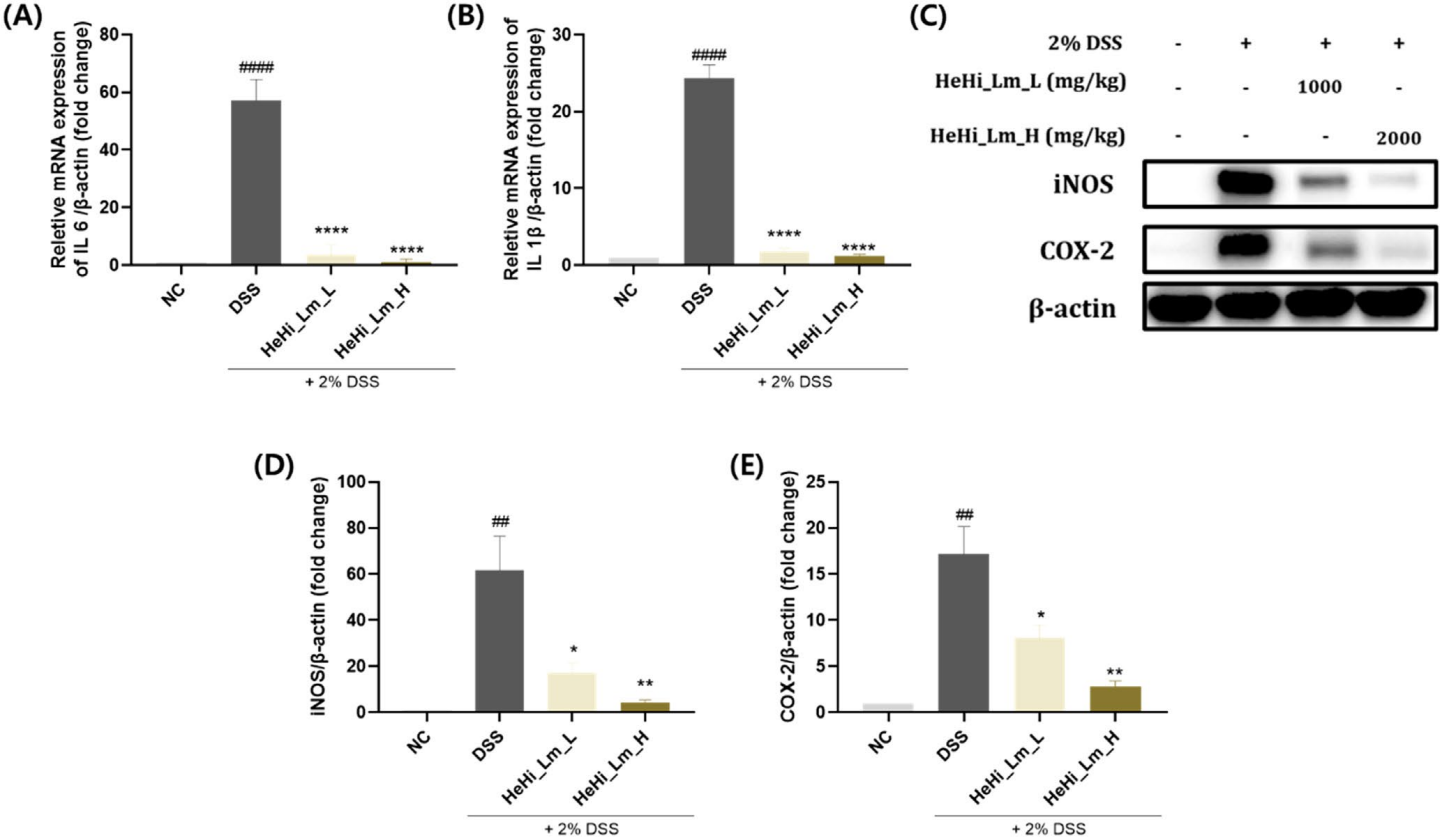
Effects of HeHi_Lm treatment on inflammatory mediators and cytokine expression in colon tissue. (A, B) Relative mRNA expression levels of IL‐6 and IL‐1β were determined by quantitative PCR (qPCR). Data are presented as mean ± SD (*n* = 3 independent biological replicates). (C) Representative western blot images of iNOS and COX‐2. (D, E) Corresponding densitometric analyses of iNOS and COX‐2. Western blotting was performed twice independently under identical conditions, and densitometric values represent the average of two technical replicates without statistical analysis. ^####^ *p* < 0.0001 compared with the normal control group (NC); ****p < 0.0001 compared with the DSS control group.

To evaluate the effects of HeHi_Lm on inflammatory cytokine expression in colon tissue, the mRNA levels of IL‐6 and IL‐1β were first analyzed by quantitative real‐time PCR. The expression levels of both IL‐6 and IL‐1β were significantly elevated in the DSS control group compared with those in the normal control (NC) group (Figure 6A,B). Administration of HeHi_Lm significantly reduced the mRNA expression of IL‐6 and IL‐1β in both the low‐dose (HeHi_Lm_L) and high‐dose (HeHi_Lm_H) groups compared with the DSS group, with expression levels approaching those observed in the NC group. Consistent with the gene expression results, Western blot analysis showed that the protein expression levels of inducible nitric oxide synthase (iNOS) and cyclooxygenase‐2 (COX‐2), key inflammatory mediators, were markedly increased in the DSS group compared with the NC group (Figure 6C–E). Treatment with HeHi_Lm significantly suppressed the DSS‐induced upregulation of iNOS and COX‐2 protein expression.

### 3.8 Effect of HeHi_Lm Treatment on Gut Microbiota Composition

**FIGURE 7:**
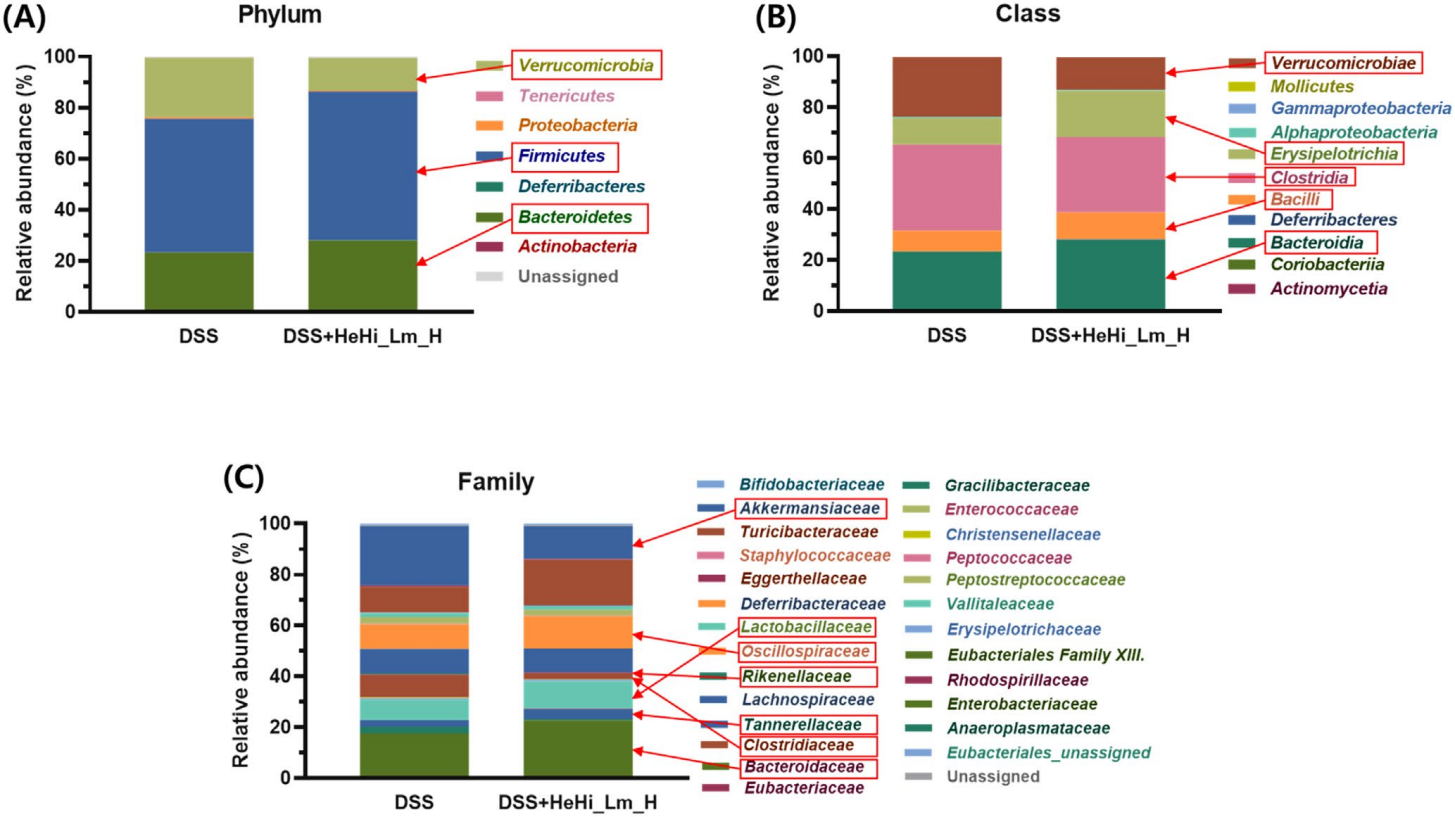
Gut microbiota composition at different taxonomic levels following HeHi_Lm_H treatment. Stacked bar plots show the relative abundance of gut microbiota at the (A) phylum, (B) class, and (C) family levels in DSS and DSS + HeHi_Lm_H groups. Bacterial taxa highlighted with red arrows indicate microbial groups specifically referenced in the text. For each group, one pooled fecal sample was used for microbiota analysis due to limited sample availability in colitis mice; therefore, these data are descriptive and no statistical analysis was performed.

Relative abundance analysis at the phylum, class, and family levels revealed clear compositional differences between the DSS and DSS + HeHi_Lm_H groups (Figure 7A–C). At the phylum level, the gut microbiota of DSS‐treated mice was primarily composed of *Firmicutes*, *Bacteroidetes*, and *Verrucomicrobia*. HeHi_Lm_H treatment increased the relative abundance of *Firmicutes* (52.38%–58.34%) and *Bacteroidetes* (22.61%–27.26%) while markedly reducing *Verrucomicrobia* (23.41%–12.71%). Minor phyla, including Actinobacteria and Proteobacteria, remained at low levels in both groups. At the class level, the increase in Bacteroidetes was mainly associated with expansion of *Bacteroidia*. Within *Firmicutes*, *Bacilli* and *Erysipelotrichia* increased, whereas *Clostridia* showed a moderate decrease. The reduction in *Verrucomicrobia* was reflected by a corresponding decrease in *Verrucomicrobiae*. At the family level, HeHi_Lm_H treatment increased Bacteroidaceae, Tannerellaceae, Lactobacillaceae, and Oscillospiraceae, while *Rikenellaceae*, *Clostridiaceae*, and *Akkermansiaceae* were reduced. Overall, these results indicate that HeHi_Lm_H treatment was associated with coordinated shifts in gut microbiota composition across multiple taxonomic levels.

## 4 Discussion

### 4.1 Therapeutic Relevance of HeHi_Lm in Inflammatory Bowel Disease

IBDs are characterized by a high recurrence rate and remain chronic inflammatory disorders for which curative treatments are currently limited. Although not typically life‐threatening, long‐term inflammation is associated with impaired quality of life and an increased risk of colorectal cancer development (Triantafillidis et al. 2009; Fanizza et al. 2024). Therefore, effective management of IBD is important for improving patient well‐being and reducing long‐term disease‐associated complications. In this study, we examined the effects of HeHi_Lm treatment on DSS‐induced chronic colitis in mice, a widely used experimental model that reflects key pathological features of human IBD.

### 4.2 Sustainability‐Driven Fermentation Strategy and Functional Metabolite Enrichment

The rationale for combining *H. illucens* with *L. mesenteroides* was based on sustainability considerations and synergistic functional effects relevant to the concept of novel foods. *H. illucens* serves as a sustainable, nutrient‐dense insect‐derived raw material, while *L. mesenteroides* is a functional lactic acid bacterium capable of enhancing the nutritional and bioactive properties of fermented substrates. Accordingly, HeHi_Lm was developed by fermenting HeHi with *L. mesenteroides* KCCM13067P under previously described fermentation conditions (Kim et al. 2026; Son et al. 2023), resulting in significant increases in riboflavin, FMN, and FAD, as well as a substantial enrichment of total free amino acids and amines compared with the unfermented material (Figure 1). Notably, fermentation increased several bioactive amino acids and amines, including glycine, alanine, β‐alanine, cysthionine, α‐aminobutyric acid, β‐aminoisobutyric acid, γ‐aminobutyric acid, and ethanolamine, indicating enhanced production of low‐molecular‐weight nitrogenous metabolites. In addition, a modest increase in total protein content was observed following fermentation, suggesting partial microbial bioconversion and improved availability of nitrogenous components within the fermented substrate. Riboflavin is metabolized in vivo into the active coenzymes FMN and FAD, which play essential roles in energy metabolism and act as potent antioxidants that protect cells from oxidative damage. Consistent with these biochemical functions, the fermentation‐driven enrichment of riboflavin and related metabolites, together with increased levels of free amino acids, amines, and protein‐derived components, was associated with enhanced anti‐inflammatory effects, supporting a synergistic interaction between the insect‐derived substrate and the LAB strain (Świder et al. 2024; Mu et al. 2025). Consequently, these fermentation‐derived metabolites are expected to collectively contribute to the protection of colon tissue, suppression of DSS‐induced inflammation, and normalization of intestinal microbiota following HeHi_Lm ingestion. These findings suggest that the anti‐inflammatory effects observed in this study cannot be attributed to riboflavin alone, but rather result from the combined actions of multiple fermentation‐derived metabolites produced during *L. mesenteroides* fermentation.

### 4.3 Anti‐Inflammatory Effects of HeHi_Lm in LPS‐Stimulated Macrophages

To investigate the anti‐inflammatory potential of HeHi_Lm at the cellular level, we evaluated its effects on nitric oxide (NO) and pro‐inflammatory cytokine production in LPS‐stimulated RAW 264.7 macrophages. LPS activates toll‐like receptor 4 (TLR4), triggering downstream inflammatory signaling pathways and promoting the production of inflammatory mediators, including NO, TNF‐α, and IL‐6 (Tatiya‐Aphiradee et al. 2018). In this model, LPS markedly increased NO and cytokine secretion, whereas pretreatment with HeHi_Lm attenuated these responses in a concentration‐dependent manner. In particular, significant suppression of NO and IL‐6 production was observed, supporting the anti‐inflammatory activity of the fermented extract. These findings suggest that HeHi_Lm modulates macrophage‐mediated inflammatory signaling and may reduce excessive inflammatory mediator release. However, RAW 264.7 cell viability showed a modest, concentration‐dependent decrease at higher doses (approximately 74%–83% of control); therefore, the magnitude of inhibition observed at 400–800 μg/mL should be interpreted cautiously, as part of the apparent reduction in inflammatory markers may partially reflect reduced cellular metabolic activity rather than purely anti‐inflammatory effects.

### 4.4 Protective Effects of HeHi_Lm in a DSS‐Induced Chronic Colitis Mouse Model

Since the DSS‐induced mouse model shows clinical symptoms very similar to those of IBD, it has been used as a tool in numerous studies to develop therapeutic agents (Jurjus et al. 2004). In particular, the mouse DSS‐induced model showed body weight loss, diarrhea, and hematochezic feces. We explored whether HeHi_Lm, which has been confirmed to have anti‐inflammatory effects in LPS‐induced RAW 264.7 cells, has similar effects in the in vivo gut inflammatory model. The biochemical and physiological processes of chronic colitis were evaluated using a DSS‐induced chronic colitis murine model because they provide a broad overview of potential treatment approaches (Jurjus et al. 2004; Li et al. 2018). Changes in body weight, diarrhea, and bloody feces were used to determine the DAI score and evaluate clinical symptoms. Additionally, after the experiment, the colon—directly related to intestinal inflammation—was excised, and its length was measured. Moreover, damage or the inflammatory reactions of colonic tissue were assessed by H&E staining for pathophysiological evaluation. The spleen, liver, and organs related to inflammation were excised, and their weights were compared. As predicted, our results revealed that administration of HeHi_Lm could have a potentially protective effect on intestinal inflammation by DSS. This was reflected by the observed changes in clinical symptoms and pathophysiology, including improvement in DAI scores and alleviation of the shortening of colon length and increase in spleen and liver weights. Similar to previous findings (Son et al. 2023), damage caused by intestinal inflammation, including significant weight loss and increased DAI, was observed during the second cycle. During the third cycle, clinical symptoms tended to recover at both low and high concentrations in the HeHi_Lm group, especially at the end of the experiment. At high concentrations, both weight changes and DAI scores were statistically significant, while at low concentrations, only weight changes showed statistical significance. Moreover, colon tissue specimen analysis using H&E staining showed that the colon tissue damage observed in the DSS group was significantly ameliorated in both HeHi_Lm (L and H) groups. In colitis, swelling occurs between the intestinal mucosal tissue, and the infiltration of inflammatory cells into the large intestine is increased. In addition, ulcers and thrombosis occur, and the crypts collapse, damaging the intestinal epithelium. Following HeHi_Lm treatment, the swelling and infiltration of inflammatory cells decreased, resulting in crypt conditions that closely resembled normal levels, and histological damage was ameliorated. Colon tissue length measurement, an indicator of inflammation, demonstrated an increasing trend at low concentrations and a statistically significant result at high concentrations. Furthermore, with the identification of another inflammatory phenotypic marker, significant reductions were observed in the spleen and liver weight/body weight ratios. This confirmed an anti‐inflammatory effect following HeHi_Lm administration at both concentrations. This inhibition of colorectal tissue contraction and reduction in spleen and liver organ weight ratios is consistent with the results of previous studies (Wang et al. 2022; Yang et al. 2019). Consequently, it can be concluded that HeHi_Lm treatment has a preventive effect on the clinical symptoms of DSS‐induced chronic colitis.

### 4.5 Regulation of Inflammatory Mediators and Cytokines by HeHi_Lm in DSS‐Induced Colitis

Pro‐inflammatory cytokines, including IL‐6 and IL‐1β, are rapidly overproduced when intestinal epithelial tissue is necrotized by inflammatory substances such as DSS, inducing an abnormal intestinal environment (Park et al. 2017; Sun et al. 2020). COX‐2 and iNOS are inducible enzymes predominantly expressed at the site of the inflammatory response. The expression of COX‐2 induces the overexpression of iNOS and prostaglandins, triggering an inflammatory response via the production of pro‐inflammatory cytokines that may contribute to intestinal damage (Sakthivel and Guruvayoorappan 2013). Additionally, p38 mitogen‐activated protein kinase is a key factor in the modulation of pro‐inflammatory cytokines, such as IL‐6 and IL‐1β, in mucosal tissue damage in patients with IBD. Here, we investigated the expression of inflammatory indicators and pro‐inflammatory cytokines in mice with DSS‐induced colitis. The levels of these inflammatory biomarkers increased in DSS‐treated mice. As in previous studies (Sun et al. 2020; Son et al. 2023), HeHi_Lm (L and H) treatment significantly inhibited the expression of COX‐2, iNOS, IL‐6, and IL‐1β. Taken together, our results showed that HeHi_Lm regulated the inflammatory mediators (COX‐2 and iNOS) and cytokines (IL‐6 and IL‐1β) to maintain the intestinal environment, eventually alleviating histopathological damage in the colon due to DSS induction.

### 4.6 Hierarchically Coordinated Remodeling of Gut Microbiota by HeHi_Lm_H in DSS‐Induced Colitis

In this study, compositional analysis of the gut microbiota at the phylum, class, and family levels revealed that HeHi_Lm_H treatment reshaped the microbiota in DSS‐induced colitis in a coordinated manner without broadly reducing overall microbial diversity. Rather than causing nonspecific disruption, HeHi_Lm_H induced selective restructuring across taxonomic levels, reflecting changes associated with improved intestinal homeostasis. At the phylum level, HeHi_Lm_H increased *Firmicutes* and *Bacteroidetes*, and *Verrucomicrobia* significantly decreased. While *Firmicutes* and *Bacteroidetes* contribute substantially to commensal metabolic activity, overrepresentation of *Verrucomicrobia* taxa, including mucin‐degraders such as *Akkermansia*, has been linked to acute intestinal inflammation, and reductions in these taxa may indicate attenuation of inflammation‐associated dysbiosis rather than loss of beneficial diversity (Zhao et al. 2024; Liu et al. 2021). Class‐level analysis showed that these phylogenetic changes were not uniform, but *Bacteroidia* within *Bacteroides*, *Bacilli* within *Firmicutes*, and *Erysipelotricia* showed an increasing trend. Although *Clostridia* decreased relatively, this did not necessarily imply loss of beneficial fermentative bacteria, as family‐level resolution demonstrated preservation or enrichment of health‐associated families such as Oscillospiraceae (Zhou et al. 2024). Family‐level insights further clarified functional implications. Lactobacillaceae, a family frequently implicated in epithelial tight junction reinforcement and host immune modulation (Nami et al. 2025), was enriched after HeHi_Lm_H treatment, along with putative SCFA‐associated families including Oscillospiraceae (Yang et al. 2021) and the major butyrate‐producing clostridial families Lachnospiraceae and Ruminococcaceae (Fusco et al. 2023). Conversely, Clostridiaceae, often containing opportunistic or inflammation‐related taxa, was markedly reduced (Murgiano et al. 2025). Within the Bacteroidetes phylum, the concurrent increase in Bacteroidaceae and decrease in Rikenellaceae suggested a qualitative shift toward families more commonly associated with metabolic and mucosal homeostasis (Liu et al. 2024; Tavella et al. 2021). Across all taxonomic levels, the reduction of Verrucomicrobiae/Akkermansiaceae supports the notion that HeHi_Lm_H mitigates the overrepresentation of mucin‐degrading bacteria observed in inflammatory states (Zhao et al. 2024). Taken together, these results indicate that HeHi_Lm_H promotes hierarchically coherent restructuring of the gut microbiota, consistent with a shift toward a microbial community less prone to inflammation and more supportive of intestinal function.

### 4.7 Association Between Microbiota Remodeling and Suppression of Colonic Inflammation by HeHi_Lm_H

In this context, the coordinated changes in gut microbiota composition observed following HeHi_Lm_H treatment appear to be closely aligned with the concomitant suppression of inflammatory mediators in colon tissue. The enrichment of Lactobacillaceae and Oscillospiraceae, together with increases in Bacteroidaceae, coincided with marked reductions in COX‐2, iNOS, IL‐6, and IL‐1β expression, suggesting that microbial taxa associated with epithelial integrity, metabolic support, and anti‐inflammatory signaling may contribute to the attenuation of DSS‐induced inflammatory responses. Conversely, the pronounced decrease in Akkermansiaceae, Clostridiaceae, and Rikenellaceae—taxa frequently linked to mucosal disruption, excessive mucus degradation, or inflammatory dysbiosis—paralleled the normalization of inflammatory marker expression toward levels observed in non‐colitic controls. Although causality cannot be established from the present data, the parallel modulation of inflammatory biomarkers and selective microbial taxa supports a functional association between HeHi_Lm‐mediated microbiota restructuring and mitigation of colonic inflammation, thereby reinforcing the role of gut microbial balance as a key component in the anti‐inflammatory effects observed in this DSS‐induced colitis model. Although circulating cytokine levels were not measured in the present study, tissue‐level inflammatory marker analysis demonstrated consistent suppression of inflammatory responses, supporting the local anti‐inflammatory effects of HeHi_Lm in DSS‐induced colitis.

## 5 Conclusions

Fermentation of *H. illucens* with the riboflavin‐producing strain *L. mesenteroides* KCCM13067P significantly enhanced vitamin B_2_ derivatives and enriched free amino acids and bioactive amines in the fermented product (HeHi_Lm). These compositional changes were associated with reduced inflammatory responses in LPS‐stimulated RAW 264.7 macrophages and alleviation of clinical symptoms, inflammatory mediator expression, and histopathological damage in a DSS‐induced chronic colitis mouse model. HeHi_Lm treatment was also accompanied by coordinated shifts in gut microbiota composition toward taxa commonly associated with intestinal homeostasis. From a fundamental food science perspective, this study demonstrates that fermentation of insect‐derived substrates with functional lactic acid bacteria can modify metabolite profiles, including vitamin B_2_ derivatives and nitrogenous metabolites, and linking these compositional changes to anti‐inflammatory activity and gut microbiota modulation. From a food industry perspective, the findings support the development of sustainable fermentation‐based strategies using insect‐derived substrates to produce functional food ingredients with enhanced nutritional and bioactive properties. Further studies addressing fermentation process optimization, safety evaluation, and human clinical validation will be required to support the development, industrial application, and commercialization of fermented insect‐based functional food ingredients.

## Author Contributions

Conceptualization, S.‐H.L. and S.M.H.; methodology, S.J.S. and A.‐R.H.; software, M.J.S.; validation, H.N. and A.‐R.H.; formal analysis, S.J.S. and A.‐R.H.; investigation, M.J.S.; resources, S.‐H.L.; data curation, S.‐H.L.; writing – original draft preparation, S.J.S.; writing – review and editing, S.‐H.L. and H.N.; visualization, S.J.S., A.‐R.H., and S.M.H.; supervision, S.‐H.L.; project administration, S.‐H.L.; funding acquisition, S.‐H.L. All authors have read and agreed to the published version of the manuscript.

## Funding

This study was supported by grants from the Ministry of Small and Medium‐sized Enterprises (SMEs) and Startups (RS‐2025‐25458500) and the Korea Food Research Institute (E0232203).

## Conflicts of Interest

The authors declare no conflicts of interest.

## Data Availability

The data that support the findings of this study are openly available in Figshare at https://figshare.com/account/items/30197707, reference number 30197707.
